# Characterizing Diagnostic Inertia in Arterial Hypertension With a Gender Perspective in Primary Care

**DOI:** 10.3389/fcvm.2022.874764

**Published:** 2022-06-16

**Authors:** Vicente Pallares-Carratala, Concepcion Carratala-Munuera, Adriana Lopez-Pineda, Jose Antonio Quesada, Vicente Gil-Guillen, Domingo Orozco-Beltran, Jose L. Alfonso-Sanchez, Jorge Navarro-Perez, Jose M. Martin-Moreno

**Affiliations:** ^1^Health Surveillance Unit, Castellon Mutual Insurance Union, Castellón, Spain; ^2^Department of Medicine, Jaume I University, Castellón, Spain; ^3^Clinical Medicine Department, Miguel Hernandez University, Elche, Spain; ^4^Department of Preventive Medicine and Public Health, School of Medicine, University of Valencia, Valencia, Spain; ^5^Preventive Medicine Department, General Hospital of Valencia, Valencia, Spain; ^6^Biomedical Research Institute INCLIVA, Clinic University Hospital of Valencia, University of Valencia, Valencia, Spain

**Keywords:** diagnostic inertia, arterial hypertension, gender, equity, primary care

## Abstract

**Background and Objectives:**

Substantial evidence shows that diagnostic inertia leads to failure to achieve screening and diagnosis objectives for arterial hypertension (AHT). In addition, different studies suggest that the results may differ between men and women. This study aimed to evaluate the differences in diagnostic inertia in women and men attending public primary care centers, to identify potential gender biases in the clinical management of AHT.

**Study Design/Materials and Methods:**

Cross-sectional descriptive and analytical estimates were obtained nested on an epidemiological ambispective cohort study of patients aged ≥30 years who attended public primary care centers in a Spanish region in the period 2008–2012, belonging to the ESCARVAL-RISK cohort. We applied a consistent operational definition of diagnostic inertia to a registry- reflected population group of 44,221 patients with diagnosed hypertension or meeting the criteria for diagnosis (51.2% women), with a mean age of 63.4 years (62.4 years in men and 64.4 years in women).

**Results:**

Of the total population, 95.5% had a diagnosis of hypertension registered in their electronic health record. Another 1,968 patients met the inclusion criteria for diagnostic inertia of hypertension, representing 4.5% of the total population (5% of men and 3.9% of women). The factors significantly associated with inertia were younger age, normal body mass index, elevated total cholesterol, coexistence of diabetes and dyslipidemia, and treatment with oral antidiabetic drugs. Lower inertia was associated with age over 50 years, higher body mass index, normal total cholesterol, no diabetes or dyslipidemia, and treatment with lipid-lowering, antiplatelet, and anticoagulant drugs. The only gender difference in the association of factors with diagnostic inertia was found in waist circumference.

**Conclusion:**

In the ESCARVAL-RISK study population presenting registered AHT or meeting the functional diagnostic criteria for AHT, diagnostic inertia appears to be greater in men than in women.

## Introduction

Arterial hypertension (AHT) is a major modifiable risk factor for cardiovascular disease and death; its adequate control is a strategy with a high degree of evidence and population benefit (1). Given its prevalence, patients with AHT in primary health care (PHC) clinics are diverse, which means that no single therapeutic approach exists (2). The most recent AHT guidelines (3, 4) recommend lower blood pressure (BP) control targets to reduce cardiovascular events, particularly in high-risk patients.

A critical analysis of the guidelines, adapted to the context of PHC, proposes that all patients with hypertension should have a target BP of <140/90 (5). At present, only 58.5% of patients achieve these targets (6), but the more ambitious target of BP < 130/80, if well-tolerated, is potentially achievable for most hypertensive patients, especially those at high or very high cardiovascular risk (5, 6).

The European guidelines (3) specifically emphasize the need to avoid therapeutic inertia. When treatment is ineffective, a proper follow-up plan is best, with the PHC team of medical and nursing professionals detecting and correcting the possible causes of poor control (5).

Therefore, greater knowledge of clinical practice guidelines by clinicians and individualized prescription of treatment are key to avoiding inertia, which will benefit hypertensive patients and contribute to improving the health of the population. Analyzing whether this has implications according to the sex (biological factors) and gender (sociocultural factors) of patients is presently essential.

Classically, differences between sexes have been estimated in the cardiovascular (CV) area. Men are more likely to develop coronary heart disease as the first event, whereas women are more likely to have cerebrovascular disease or heart failure as the first manifestation, although these appear more frequently at advanced ages (7). Another issue is the potential inequity in clinical care that has been observed by gender, due to biases conditioned by social attributes or differences in opportunity for men and women (8). In May 2021, The Lancet Women and Cardiovascular Disease Commission published its “call to action” report including recommendations to reduce the global burden of CV disease in women by 2030. This reflects the necessity to include gender objectives in achieving CV health objectives to achieve equity in clinical care practice (9).

Despite recent progress in basic and clinical research on the differences in the management and outcomes of AHT in men and women, the main guidelines of the leading AHT societies continue not to identify gender differences due to the lack of conclusive results in clinical trials (3, 4, 10). One of the most recent and high-impact trials, the SPRINT study, failed to recruit 50% women as planned (including only 36%) and was therefore underpowered for gender analysis (11).

Subgroup analyses comparing results between women and men have been subsequently published, but they only contributed to highlight that their results were inconclusive for women and that implementation of their results concerning sex should be considered with caution (12–14). In subsequent studies, the inclusion of women has been increased, such as ACCOMPLISH (39.5%) (15), VALUE (42%) (16), and HOPE 3 (46%) (17). However, a paucity remains of sex-specific data to guide the treatment of hypertension in non-pregnant women, despite the fact that nearly 800 million women worldwide are hypertensive (18). Clearly, much work remains since the antihypertensive treatment proposed by HTA guidelines based on a gender approach may overlook sex- linked pathophysiologic and therapeutic considerations (19–22). Therefore, we consider that a gender-specific approach to hypertension prevention, diagnosis, and treatment programs should be implemented to address the more than likely gender differences to achieve more effective health promotion outcomes.

This study aimed to analyze, within the ESCARVAL-RISK study cohort, the difference in the diagnostic management and results of AHT between women and men attending public primary care centers, including those meeting the criteria for AHT diagnosis and, according to clinical practice guidelines, not properly diagnosed or treated in the PHC setting. In addition, it aimed to describe the profile of patients affected by diagnostic inertia (DI).

## Methods

This was a cross-sectional epidemiological study nested on an epidemiological ambispective cohort study carried out in the Valencian Community (an autonomous community of Spain with an estimated population of over 5 million people) in 2020 (23). This study was approved by an ethical committee and was conducted following the guidelines of the Declaration of Helsinki. The details of the protocol for this study have been previously described (24).

Patients were selected from the ESCARVAL-RISK cohort (19). This included women and men with cardiovascular risk factors (CVRF) and free of events (hospital admission for ischemic heart disease or stroke) who were seen in PHC consultations for routine clinical practice between 2008 and 2012. Baseline data were obtained from the electronic health record (EHR) of patients who met the inclusion criteria. Eligible patients were women and men aged 30 years or older and with AHT.

A patient was considered to have AHT if, during a baseline window period of 6 months from inclusion: (a) they had AHT coded in the EHR (Code I10–5 according to the International Classification of Diseases, ICD-10) and were being treated for this (pharmacological or non-pharmacological intervention) or (b) despite no diagnosis of AHT, they had been prescribed antihypertensive drugs or had two altered systolic (SBP) or diastolic (DBP) blood pressure readings (SBP ≥ 140 or DBP ≥ 90), in accordance with the criteria established by the clinical practice guidelines for the period analyzed (25, 26). Patients with inconsistent or incomplete data in their EHR were excluded.

### Study Variables

The primary variable was DI in AHT, considered operationally when a patient had two altered blood pressure readings (SBP ≥ 140 or DBP ≥ 90), as established by clinical guidelines (25, 26), during a 6-months baseline window period from inclusion, and neither the diagnosis of hypertension was coded in the EHR, nor the patient was treated with antihypertensive drugs.

Other variables studied were sociodemographic variables (age and sex/gender), clinical variables (body mass index [BMI], waist circumference, SBP, and DBP), variables related to lifestyle (smoking status), and analytical indicators (glycosylated hemoglobin [HbA1c], high-density lipoprotein cholesterol [HDL-c], triglycerides, and total cholesterol). A value was considered missing when no data existed for the variable in the EHR (≥ 50%). In addition, variables corresponding to pathologies recorded in the EHR according to the ICD-9 code were collected: diabetes mellitus (250.0), dyslipidemia (272.0), proteinuria (791.0), retinopathy (362.0), metabolic syndrome (277.7), ischemic heart disease (410.0–14.0), heart failure (428.0), peripheral artery disease (440.20), atrial fibrillation (427.31), and chronic kidney disease (585.9). Finally, variables related to medication were collected: antihypertensive treatment, lipid-lowering drugs (statins and others), oral antidiabetic drugs, insulin, and antiplatelet or anticoagulant agents.

The source of information for all the variables was the ABUCASIS EHR, which is centralized and unique for the entire Valencian Community.

### Statistical Analysis

To estimate the prevalence of DI, the number and frequency of inertia cases were calculated for the total and by sex. To evaluate the patient profile according to their DI in each category of qualitative variables, double-entry tables were made by applying the Chi-Square statistical test.

Prevalence ratios (PRs) and 95% confidence intervals (95% CIs) of inertia at each level of the explanatory variables were estimated using multivariate Poisson regression models with robust variance (27), differentiating by sex. A stepwise variable selection procedure was performed, based on the Akaike information criterion (AIC). The multicollinearity of the variables in the construction of the models was studied. The goodness-of-fit likelihood ratio test (LRT), AIC value, and receiver operating characteristic (ROC) area of each model were performed. To avoid the multiplicity problem due to the analysis by subgroups due to sex/gender, the type I error was adjusted by the Bonferroni method to 0.025. The analyses were performed using IBM SPSS Statistics for Windows, *v*. 26.0 (IBM Corporation, Armonk, NY, United States) and R software, *v*. 4.0.2 (R Core Team, Vienna, Austria).

## Results

A total of 44,221 patients with diagnosed AHT, or meeting the diagnostic criteria for AHT and undiagnosed or on antihypertensive treatment and coded in the EHR, were included (51.2% women). The mean age of the patients was 63.4 years (range 30–97 years), being 62.4 years in men (range 30–95 years) and 64.4 years in women (range 30–97 years).

A total of 1,968 patients were identified who met the DI inclusion criteria and had no coded diagnosis of AHT, representing 4.5% of the total population studied. By sex, 5% were men and 3.9% were women (*p* < 0.001; Table 1).

**Table 1:** Diagnostic inertia in hypertension for all hypertensive patients and by gender.

		**Diagnostic AHT**		**DI**		
		***n***	**%**	***n***	**%**	***p*-value**
**Gender**	Men	20,512	95.0%	1.082	5.0%	<0.001
	Women	21,741	96.1%	886	3.9%	
	Total	42,253	95.5%	1.968	4.5%	

*AHT, arterial hypertension; DI, diagnostic inertia*.

Tables 2, 3 show the analysis of DI with respect to the population diagnosed with hypertension in the sociodemographic and clinical variables and sexes. Statistically significant differences of more DI were found in men with normal BMI (*p* < 0.001) and waist circumference (*p* < 0.001), who were smokers (*p* < 0.02), with normal HDL (*p* < 0.001), and with cholesterol >200 mg/dL (*p* < 0.001). In women, the highest DI was associated with the youngest age group (*p* < 0.013), normal BMI (*p* < 0.001), and total cholesterol >200 mg/dL (*p* < 0.001).

**Table 2:** Prevalence of diagnostic inertia in hypertensive patients in sociodemographic and clinical variables (men).

	**Total**		**Diagnostic AHT**		**DI**		***p*-value**
	***n***	**%**	***n***	**%**	***n***	**%**	
**Age groups (yrs)**							
30–49	3,106	14.4%	2,935	94.5%	171	5.5%	0.409
50–59	4,932	22.8%	4,699	95.3%	233	4.7%	
60–69	7,246	33.6%	6,892	95.1%	354	4.9%	
≥70	6,310	29.2%	5,986	94.9%	324	5.1%	
**BMI** ^**a**^							
Normal	1,970	9.1%	1,814	92,1%	156	7.9%	<0.001
Overweight	8,617	39.9%	8,122	94.3%	495	5.7%	
Obesity	8,387	38.8%	8,012	95.5%	375	4.5%	
Missing	2,620	12.1%	2,564	97.9%	56	2.1%	
**Abdominal perimeter**							
Normal	4,182	19.4%	3,867	92.5%	315	7.5%	<0.001
≥88/102 cm	6,737	31.2%	6,396	94.9%	341	5.1%	
Missing	10,675	49.4%	10,249	96.0%	426	4.0%	
**Smoking status**							
No	7,867	36.4%	7,488	95.2%	379	4.8%	0.020
Si	6,028	27.9%	5,686	94.3%	342	5.7%	
Quit smoking	7,699	35.7%	7,338	95.3%	361	4.7%	
**PP**							
<40 mmHg	2,396	11.1%	2,281	952%	115	4.8%	0.910
40–60 mmHg	7,183	33.3%	6,814	94.9%	369	5.1%	
>60 mmHg	1,791	8.3%	1,700	94.9%	91	5.1%	
Missing	10,224	47.3%	9,717	95.0%	507	5.0%	
**DBP**							
Normal	7,774	36.0%	7,390	95.1%	384	4.9%	0.662
≥90 mmHg	3,596	16.7%	3,405	94.7%	191	5.3%	
Missing	10,224	47.3%	9,717	95.0%	507	5.0%	
**SBP**							
Normal	6,622	30.7%	6,290	95.0%	332	5.0%	0.917
≥140 mmHg	4,748	22.0%	4,505	94.9%	243	5.1%	
Missing	10,224	47.3%	9,717	95.0%	507	5.0%	
**HDL-Cholesterol**							
Normal	5,677	26.3%	5,348	94.2%	329	5.8%	<0.001
≤ 45 mg/dL	5,328	24.7%	5,044	94.7%	284	5.3%	
Missing	10,589	49.0%	10,120	95.6%	469	4.4%	
**Total cholesterol**							
Normal	5,727	26.5%	5,465	95.4%	262	4.6%	<0.001
≥200 mg/dL	5,973	27.7%	5,579	93.4%	394	6.6%	
Missing	9,894	45.8%	9,468	95.7%	426	4.3%	

*AHT, arterial hypertension; DI, diagnostic inertia; BMI, body mass index; PP, pulse pressure; DBP, diastolic blood pressure; SBP, diastolic blood pressure; HDL, high density lipoprotein*.

*^a^Normal <25 kg/m^2^; overweight 25.0–29.9 kg/m^2^; obese ≥30.0 kg/m^2^*.

**Table 3:** Prevalence of diagnostic inertia in hypertensive patients in sociodemographic and clinical variables (women).

	**Total**		**Diagnostic AHT**		**DI**		***p*-value**
	***n***	**%**	***n***	**%**	***n***	**%**	
**Age groups (yrs)**							
30–49	2,449	10.8%	2,324	94.9%	125	5.1%	0.013
50–59	4,719	20.9%	4,547	96.4%	172	3.6%	
60–69	7,297	32.2%	7,013	96.1%	284	3.9%	
≥70	8,162	36.1%	7,857	96.3%	305	3.7%	
**BMI** ^**a**^							
Normal	2,700	11.9%	2,550	94.4%	150	5.6%	<0.001
Overweight	7,274	32.1%	6,947	95.5%	327	4.5%	
Obesity	9,948	44.0%	9,607	96.6%	341	3.4%	
Missing	2,705	12.0%	2,637	97.5%	68	2.5%	
**Abdominal perimeter**							
Normal	1,431	6.3%	1,377	96.2%	54	3.8%	0.051
≥88/102	10,109	44.7%	9,678	95.7%	431	4.3%	
Missing	11,087	49.0%	10,686	96.4%	401	3.6%	
**Smoking status**							
No	18,192	80.4%	17,467	96.0%	725	4.0%	0.398
Si	2,950	13.0%	2,838	96.2%	112	3.8%	
Quit smoking	1,485	6.6%	1,436	96.7%	49	3.3%	
**PP**							
<40	2,532	11.2%	2,428	95.9%	104	4.1%	0.957
40–60	7,512	33.2%	7,220	96.1%	292	3.9%	
>60	1,913	8.5%	1,840	96.2%	73	3.8%	
Missing	10,670	47.2%	10,253	96.1%	417	3.9%	
**DBP**							
Normal	8,158	36.1%	7,834	96.0%	324	4.0%	0.919
≥90	3,799	16.8%	3,654	96.2%	145	3.8%	
Missing	10,670	47.2%	10,253	96.1%	417	3.9%	
**SBP**							
Normal	6,860	30.3%	6,584	96.0%	276	4.0%	0.803
≥140	5,097	22.5%	4,904	96.2%	193	3.8%	
Missing	10,670	47.2%	10,253	96.1%	417	3.9%	
**HDL-Cholesterol**
Normal	9,164	40.5%	8,774	95.7%	390	4.3%	0.080
≤ 45	2,395	10.6%	2,302	96.1%	93	3.9%	
Missing	11,068	48.9%	10,665	96.4%	403	3.6%	
**Total cholesterol**							
Normal	4,755	21.0%	4,607	96.9%	148	3.1%	<0.001
>200	7,485	33.1%	7,123	95.2%	362	4.8%	
Missing	10,387	45.9%	10,011	96.4%	376	3.6%	

*AHT, arterial hypertension; DI, diagnostic inertia; BMI, body mass index; PP, pulse pressure; DBP, diastolic blood pressure; SBP, diastolic blood pressure; HDL, high density lipoprotein*.

*^a^Normal <25 kg/m^2^; overweight 25.0–29.9 kg/m^2^; obese ≥ 30.0 kg/m^2^*.

Tables 4, 5 show the analysis of DI according to comorbidities and treatments in men and women, respectively. Statistically significant differences and higher DI were found in men without heart failure (*p* < 0.028) or peripheral artery disease (*p* < 0.001), with diabetes (*p* < 0.001) and dyslipidemia (*p* < 0.016), on oral antidiabetic treatment (*p* < 0.001), and not taking lipid-lowering, antiplatelet, or anticoagulant treatment (*p* < 0.001). In women, the highest DI was observed in those with diabetes and dyslipidemia (*p* < 0.001), without heart failure (*p* < 0.018), treated with oral antidiabetic drugs (*p* < 0.001), and not on antiplatelet or anticoagulant therapy (*p* < 0.001).

**Table 4:** Prevalence of diagnostic inertia in hypertensive patients with comorbidity and treatments (men).

	**Total**		**Diagnostic AHT**		**DI**		***p*-value**
	***n***	**%**	***n***	**%**	***n***	**%**	
**Heart failure**							
No	21,286	98.6%	20,213	95.0%	1,073	5.0%	0.028
Yes	306	1.4%	299	97.7%	7	2.3%	
**Proteinuria**							
No	21,449	99.3%	20,372	95.0%	1,077	5.0%	0.387
Yes	145	0.7%	140	96.6%	5	3.4%	
**PAD**							
No	21,084	97.7%	20,015	94.9%	1,069	5.1%	0.001
Yes	506	2.3%	497	98.2%	9	1.8%	
**Atrial fibrillation**							
No	21,413	99.2%	20,338	95.0%	1,075	5.0%	0.173
Yes	179	0.8%	174	97.2%	5	2.8%	
**Diabetes mellitus**							
No	14,537	67.3%	14,100	97.0%	437	3.0%	<0.001
Yes	7,057	32.7%	6,412	90.9%	645	9.1%	
**Dyslipidemia**							
No	10,470	48.5%	9,984	95.4%	486	4.6%	0.016
Yes	11,124	51.5%	10,528	94.6%	596	5.4%	
**CKD**							
No	21,375	99.0%	20,300	95.0%	1,075	5.0%	0.067
Yes	217	1.0%	212	97.7%	5	2.3%	
**Retinopathy**							
No	21,484	99.5%	20,408	95.0%	1,076	5.0%	0.831
Yes	110	0.5%	104	94.5%	6	5.5%	
**Insulin**							
No	21,117	97.8%	20,060	95.0%	1,057	5.0%	0.816
Yes	477	2.2%	452	94.8%	25	5.2%	
**Oral antidiabetics**							
No	18,519	85.8%	17,686	95.5%	833	4.5%	<0.001
Yes	3,075	14.2%	2,826	91.9%	249	8.1%	
**Lipid-lowering**							
No	16,326	75.6%	15,445	94.6%	881	5.4%	<0.001
Yes	5,268	24.4%	5,067	96.2%	201	3.8%	
**Antiplatelet agents**							
No	17,952	83.1%	16,950	94.4%	1,002	5.6%	<0.001
Yes	3,642	16.9%	3,562	97.8%	80	2.2%	
**Anticoagulants**							
No	18,648	86.4%	17,646	94.6%	1,002	5.4%	<0.001
Yes	2,946	13.6%	2,866	97.3%	80	2.7%	

*AHT, arterial hypertension; DI, diagnostic inertia; PAD, peripheral artery disease; CKD, chronic kidney disease*.

**Table 5:** Prevalence of diagnostic inertia in hypertensive patients with comorbidity and treatments (women).

	**Total**		**Diagnostic AHT**		**DI**		***p*-value**
	***n***	**%**	***n***	**%**	***n***	**%**	
**Heart failure**							
No	22,211	98.2%	21,333	96.0%	878	4.0%	0.018
Yes	415	1.8%	408	98.3%	7	1.7%	
**Proteinuria**							
No	22,520	99.5%	21,639	96.1%	881	3.9%	0.578
Yes	105	0.5%	102	97.1%	3	2.9%	
**PAD**							
No	22,478	99.3%	21,596	96.1%	882	3.9%	0.235
Yes	148	0.7%	145	98.0%	3	2.0%	
**Atrial fibrillation**
No	22,496	99.4%	21,612	96.1%	884	3.9%	0.064
Yes	130	0.6%	129	99.2%	1	0.8%	
**Diabetes mellitus**	
No	16,511	73.0%	16,012	97.0%	499	3.0%	<0.001
Yes	6,116	27.0%	5,729	93.7%	387	6.3%	
**Dyslipidemia**							
No	10,313	45.6%	9,995	96.9%	318	3.1%	<0.001
Yes	12,314	54.4%	11,746	95.4%	568	4.6%	
**CKD**							
No	22,486	99.4%	21,603	96.1%	883	3.9%	0.052
Yes	139	0.6%	138	99.3%	1	0.7%	
**Retinopathy**							
No	22,522	99.5%	21,638	96.1%	884	3.9%	0.287
Yes	105	0.5%	103	98.1%	2	1.9%	
**Insulin**							
No	22,042	97.4%	21,178	96.1%	864	3.9%	0.845
Yes	585	2.6%	563	96.2%	22	3.8%	
**Oral antidiabetics**							
No	19,995	88.4%	19,267	96.4%	728	3.6%	<0.001
Yes	2,632	11.6%	2,474	94.0%	158	6.0%	
**Lipid-lowering**							
No	17,154	75.8%	16,459	95.9%	695	4.1%	0.062
Yes	5,473	24.2%	5,282	96.5%	191	3.5%	
**Antiplatelet agents**							
No	17,053	75.4%	16,261	95.4%	792	4.6%	<0.001
Yes	5,574	24.6%	5,480	98.3%	94	1.7%	
**Anticoagulants**							
No	20,633	91.2%	19,791	95.9%	842	4.1%	<0.001
Yes	1,994	8.8%	1,950	97.8%	44	2.2%	

*AHT, arterial hypertension; DI, diagnostic inertia; PAD, peripheral artery disease; CKD, chronic kidney disease*.

**Figure 1:**
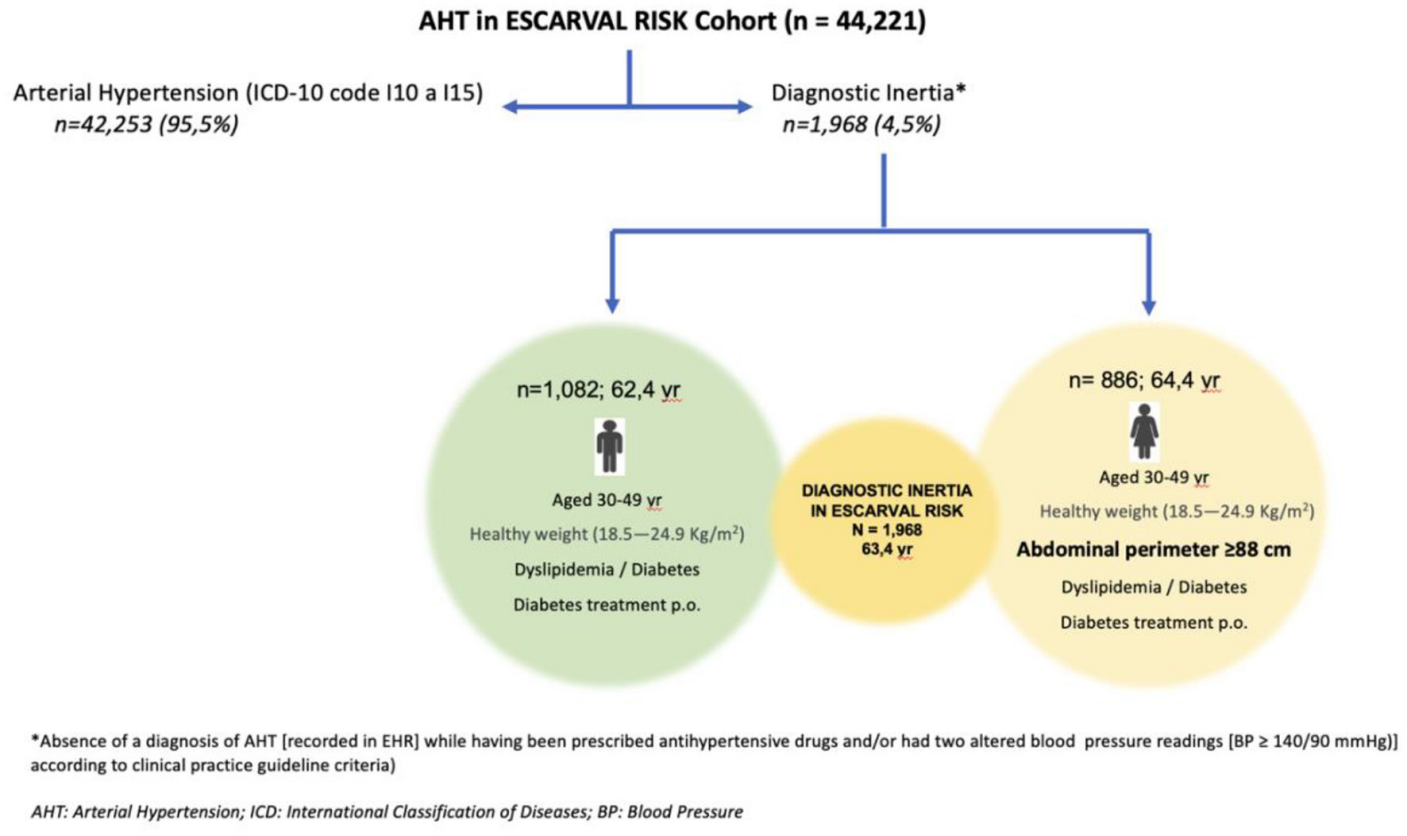
Associated factors with diagnostic inertia in hypertensive patients according to gender in the ESCARVAL RISK Cohort. * Absence of a diagnosis of AHT (recorded in EHR) while having been prescribed antihypertensive drugs and/or had two altered blood pressure readings (BP ≥ 140/90 mmHg) according to clinical practice guideline criteria. AHT, arterial hypertension; ICD, International Classification of Disease; BP, blood pressure.

Table 6 shows the PRs of DI in AHT, estimated by multivariate Poisson regression models. One model was fitted for men and another for women. The statistically significant factors associated with DI were age, BMI, waist circumference, total cholesterol, diabetes, dyslipidemia, lipid-lowering treatment, oral antidiabetic drugs, and antiplatelet and anticoagulant treatments. The observed pattern of DI between men and women was similar, except for waist circumference, where a waist circumference ≥102 cm in men was associated with lower DI, whereas a waist circumference ≥88 cm in women was associated with higher DI (Figure 1). Additionally, treatment for dyslipidemia was associated with lower DI in men and not associated in women. The model sample size was 21,594 with 1,082 cases of DI in men, and 22,627 with 886 cases of DI in women. Both models fit the data well (LRT *p* < 0.001) and presented adequate classification indicators (ROC area 0.72 and 0.69, respectively).

**Table 6:** Multivariable Poisson regression, prevalence ratios (PRs) for diagnostic inertia, by sex.

		**Men**			**Women**		
		**PR**	**(95% CI)**	***p* value**	**PR**	**(95% CI)**	***p* value**
Age groups (yrs)	30–49	1			1		
	50–59	0.72	(0.59–0.87)	0.001	0.64	(0.51–0.80)	<0.001
	60–69	0.73	(0.61–0.87)	<0.001	0.66	(0.53–0.81)	<0.001
	≥70	0.79	(0.66–0.95)	0.013	0.64	(0.52–0.79)	<0.001
Body mass index^a^	Normal	1			1		
	Overweight	0.71	(0.60–0.84)	<0.001	0.72	(0.59–0.88)	0.001
	Obese	0.54	(0.45–0.66)	<0.001	0.52	(0.42–0.63)	<0.001
	Missing	0.35	(0.26–0.48)	<0.001	0.45	(0.34–0.60)	<0.001
Waist	<88/102	1			1		
circumference	≥88/102	0.83	(0.7–0.98)	0.025	1.43	(1.06–1.92)	0.018
	Missing	0.72	(0.62–0.83)	<0.001	1.31	(0.98–1.76)	0.066
Total cholesterol^b^	Normal	1			1		
	Elevated	1.54	(1.32–1.79)	<0.001	1.63	(1.36–1.97)	<0.001
	Missing	1.09	(0.94–1.26)	0.252	1.35	(1.12–1.62)	0.001
Comorbidities	Diabetes	3.17	(2.77–3.62)	<0.001	2.24	(1.91–2.61)	<0.001
	Dislipemia	1.20	(1.06–1.36)	0.003	1.47	(1.29–1.68)	<0.001
Treatments	Antiplatelets	0.45	(0.36–0.56)	<0.001	0.38	(0.30–0.47)	<0.001
	Oral antidiabetics	1.24	(1.06–1.46)	0.008	1.25	(1.02–1.52)	0.028
	Antithrombotics	0.51	(0.40–0.64)	<0.001	0.53	(0.39–0.72)	<0.001
	TTO DLP	0.74	(0.62–0.88)	0.001			
N		21,594			22,627	
N with diagnostic inertia		1,082			886	
LRT (*p* value)	656	(<0.001)		384	(<0,001)	
AIC		8,020			7,161	
Area under the ROC (95% CI)	0.727	(0.712–0.742)		0.688	(0.672–0.705)	

*AIC, akaike information criterion; CI, confidence interval; LRT, likelihood ratio test*.

*^a^Normal <05 kg/m^2^; overweight 25.0–29.9 kg/m^2^; obese ≥ 30.0 kg/m^2^*.

*^b^Normal ≤ 200 mg/dL, elevated > 200 mg/dL*.

## Discussion

This study aimed to shed light on the magnitude of DI in the study population, that is, individuals who were undiagnosed and untreated for arterial hypertension despite meeting the criteria that should have led to being diagnosed and adequately treated for this condition. The highest DI occurred in the population aged 30–49 years, with normal BMI, elevated cholesterol (≥ 200 mg/dl), coexistence of diabetes and dyslipidemia, and taking oral antidiabetic treatment. Gender differences in DI were detected in women with a waist circumference ≥ 88 cm. In the population of the ESCARVAL-RISK cohort with AHT criteria, the percentage was 4.5%, being higher in men than in women (5 vs. 3.9%; *p* < 0.001). Although this may seem low, the great difference from other studies is that the denominator in our study is not the entire population but only those with hypertension or meeting diagnostic criteria. Therefore, these figures are particularly relevant from the clinical viewpoint.

The increase in the prevalence of chronic diseases, including AHT, is a worldwide problem with multifactorial and complex causes (3–5). Despite the increasing knowledge in the field of AHT, its high prevalence, the low degree of control, the associated morbidity and mortality, the associated costs, and the rates of non-compliance with both lifestyle measures and pharmacological treatment continue to be alarming (2–6, 18). In a PHC setting and a hypertensive population, 4.5% of the adults who met the diagnostic criteria for AHT had no recorded diagnosis or treatment prescribed. The patients who presented more DI were younger, had a normal weight, and took oral antidiabetic drugs (diabetes confers a higher CV risk), a pattern that differed slightly between men and women. In addition, many clinical variables not recorded in the EHR were detected. In a previous study of the same cohort but a dyslipidemic population, 18% of adults met the diagnostic criteria for dyslipidemia and had no recorded diagnosis or treatment prescribed (DI), with DI greater in women, young age, normal weight, no smoking habit, and those with alterations in SBP, HDL cholesterol, total cholesterol, LDL cholesterol, or triglycerides, or missing values in their EHR (28). A similar pattern exists between both sexes, and in DI in both hypertension and dyslipidemia, there is a lack of assessment of subclinical disease (comorbidities). This may promote clinical and therapeutic inertia and determine a different course in the continuum of cardiovascular disease.

A diagnosis of AHT and older age (>70 years) had a greater association with DI in men than in women, in contrast to the results observed by Meador et al. (29) in the USA, where younger, white, healthy-weight women were less likely to be diagnosed. In a previous study analyzing only patients with BP recordings ≥140/90 mmHg on EHR and no decision made (i.e., DI), the association of inertia was higher in men and older age (30).

Gil-Guillén et al. (31) observed a higher level of inertia in women with hypertension, and an association between inertia and not smoking, which in our study was only observed in men (*p* < 0.02). Ji et al. (32) analyzed sex differences in hypertension and observed that, although the prevalence was higher in men than women in the younger and middle ages of life, this reversed after the seventh decade, when the rates in women exceeded those in men. These higher BP levels in older women were associated with a higher risk of stroke than in men of the same age group.

Notably, the increased risk of stroke with higher BP levels seemed to be almost twice as high in women as in men (9). The DI detected could be a reason for an increased risk of stroke in our population. All these findings indicate the need to continue exploring possible biases or other factors, not specifically clinical, in the “non-diagnosis” of AHT (33).

Precedent exists for different strategies to improve intervention at the health care system level to reduce inertia (34). The EHR quality improvement initiative of Kaiser Permanente of Northern California, reaching more than 650,000 patients within its hypertension registry program (35), was focused on creating a registry of reporting AHT control rates (every 1–3 months) by each affiliated medical center and generating clinical practice guideline recommendations on a case-by-case basis. The effort led to improving the BP control rate in their hypertensive population to 80%, compared with a national average of 64% (36). If this were applied to our system, we are convinced of the potential improvement of inertia, not only therapeutic but also diagnostic, given the high accessibility of healthcare in our system that makes it possible to screen the population (37).

In this study, we have analyzed a hypertensive population according to sex/gender, observing many possibilities for improvement in diagnostic confirmation. We have a great capacity for improvement in DI, as long as the possible solutions contemplate three domains: health professionals, patients, and governmental agencies; promoting active health policies; and improving the tools with which the family physician currently works.

### Strengths and Limitations

The great strength of this study is its information source, which corresponds to a single electronic health registry that integrates all the information on the population attending primary care centers. In addition, it exhaustively addresses the important problem of DI in AHT and its gender differences, with a large sample size, which minimizes random error when drawing conclusions from the results obtained. The fact that the information was obtained from all the PHC centers, and that it quantifies the problem of DI in all the professionals working in these centers, offers greater validity to our results and means that they can be generalized to other geographical areas with similar healthcare systems. Therefore, it would be interesting to carry out similar studies in other countries with different health policies through projects that could integrate many patients and health professionals.

The main theoretical limitation of this study is that, although it works on the basis of an epidemiological cohort study, its strict design is cross-sectional. Establishing a temporal sequence between the factors and the dependent variable (inertia) is not realizable, although the status of undiagnosed hypertensive patients can be assessed and their needs determined, which are key elements in the fight against the lack of awareness of this problem and provide a basis for prioritizing better health planning. We are aware that the main potential bias that could have threatened the validity of this study is in selection, but we have tried to minimize this. Furthermore, the virtue of the study is that it translates routine clinical practice and is based on the fact that these are all patients attending PHC centers. We must also ask ourselves about the precise quality of the data obtained from the EHR and entered by the health professionals. To minimize this potential error, all physicians have previously been given the opportunity to participate in courses on cardiovascular risk (online, voluntary, and free), providing training on cardiovascular diseases, their risk factors, and their control objectives (38). Additionally, the service provider (*Conseller*í*a de Sanitat de la Generalitat Valenciana, Valencian Community, Spain*) has made efforts to ensure that all primary care offices had validated and calibrated BP measurement devices.

### Clinical Implications

Physicians attending PHC consultations should be attentive to BP values ≥140/90 mmHg, verify them, confirm them, and record them in the EHR, in addition to properly coding patients who are already on antihypertensive treatment to reduce the DI in AHT. Although it should be all patients, special attention should be paid to young women who are not properly identified, thus avoiding possible health inequalities derived from DI. In our study, the overall DI was higher in men than in women; this difference may be due to the type of population more likely to seek consultation in PHC, corresponding to women and older patients. The information provided by this study could be valuable for improving clinical practice in the PHC setting and could favor future research to explore the reasons for the conservative attitude of PHC physicians regarding this type of patient. Future studies should address the causes of the gender difference in the prevalence of DI in AHT and whether it affects other entities that increase CV morbidity and mortality. Therefore, the strategy should be comprehensive and close any knowledge gap, optimizing the diagnosis and control of AHT at a global level.

## Conclusions

When comparing the population diagnosed with AHT with the population not diagnosed but presenting diagnostic criteria, the highest DI (in both men and women) was in the population aged 30–49 years, with normal BMI, high cholesterol, and coexistence of diabetes and dyslipidemia, and taking oral antidiabetic treatment. The lowest DI was in the population over 50 years of age, with overweight or obesity by BMI, normal cholesterol, no diabetes or dyslipidemia, and taking antiplatelet, anticoagulant, or lipid- lowering therapy. The only gender difference in this study was found in waist circumference. In women, a greater DI was found from 88 cm, and in men, the higher the BMI, the lower the DI.

Although AHT is simple to diagnose and relatively easy to treat with currently low-cost drugs (plus healthy lifestyle recommendations), important gaps exist in the diagnosis that can have a negative impact on prognosis and evolution, which should be identified and addressed.

## Data Availability Statement

The data analyzed in this study is subject to the following licenses/restrictions: data on all patients registered in Primary Care of the Health System of the Valencian Community in Spain. The data were anonymised after a rigorous validation process, and are subject to personal data protection regulations. Requests to access these datasets should be directed to Conselleria de Sanitat Universal i Salut Pública, psalud_val@gva.es.

## Ethics Statement

The studies involving human participants were reviewed and approved by Ethics Committee of the University of Valencia Hospital Clinic, Valencia, Spain. The patients/participants provided their written informed consent to participate in this study.

## Author Contributions

Conceptualization, methodology, writing—review and editing, and funding acquisition: VP-C, CC-M, AL-P, JQ, VG-G, DO-B, JA-S, JN-P, and JM-M. Writing—original draft preparation and supervision: VP-C, CC-M, VG-G, and JM-M. Project administration: JM-M. All authors have read and agreed to the published version of the manuscript.

## Funding

The authors acknowledge support from the Health Research Projects—Strategic Action in Health (Reference: PI18/01937) of the Spanish *Fondo de Investigación Sanitaria—Instituto de Salud Carlos III*, co-funded by the European Regional Development Fund/European Social Fund: A way to make Europe/Investing in Your Future. This funding source had no role in the design of the study, its execution and analyses, the interpretation of the data, or the decision to submit results.

## Conflict of Interest

The authors declare that the research was conducted in the absence of any commercial or financial relationships that could be construed as a potential conflict of interest.

## Publisher's Note

All claims expressed in this article are solely those of the authors and do not necessarily represent those of their affiliated organizations, or those of the publisher, the editors and the reviewers. Any product that may be evaluated in this article, or claim that may be made by its manufacturer, is not guaranteed or endorsed by the publisher.
